# Genetic Predictions of Prion Disease Susceptibility in Carnivore Species Based on Variability of the Prion Gene Coding Region

**DOI:** 10.1371/journal.pone.0050623

**Published:** 2012-12-07

**Authors:** Paula Stewart, Lauren Campbell, Susan Skogtvedt, Karen A. Griffin, Jon M. Arnemo, Morten Tryland, Simon Girling, Michael W. Miller, Michael A. Tranulis, Wilfred Goldmann

**Affiliations:** 1 Neurobiology Division, The Roslin Institute & R(D)SVS, University of Edinburgh, Roslin, United Kingdom; 2 Norwegian School of Veterinary Science, Dept. Basic Sciences & Aquatic Medicine, Oslo, Norway; 3 Wildlife Research Center, Colorado Division of Parks and Wildlife, Fort Collins, Colorado, United States of America; 4 Department of Forestry & Wildlife Management, Faculty of Applied Ecology and Agricultural Sciences, Hedmark University College, Campus Evenstad, Elverum, Norway & Department of Wildlife, Fish and Environmental Studies, Faculty of Forest Sciences, Swedish University of Agricultural Sciences, Umeå, Sweden; 5 Norwegian School of Veterinary Science, Section of Arctic Veterinary Medicine, Tromsø, Norway; 6 GenØk - Centre for Biosafety, Tromsø, Norway; 7 Royal Zoological Society of Scotland, Edinburgh Zoo, Edinburgh, United Kingdom; Colorado State University, College of Veterinary Medicine and Biomedical Sciences, United States of America

## Abstract

Mammalian species vary widely in their apparent susceptibility to prion diseases. For example, several felid species developed prion disease (feline spongiform encephalopathy or FSE) during the bovine spongiform encephalopathy (BSE) epidemic in the United Kingdom, whereas no canine BSE cases were detected. Whether either of these or other groups of carnivore species can contract other prion diseases (e.g. chronic wasting disease or CWD) remains an open question. Variation in the host-encoded prion protein (PrP^C^) largely explains observed disease susceptibility patterns within ruminant species, and may explain interspecies differences in susceptibility as well. We sequenced and compared the open reading frame of the *PRNP* gene encoding PrP^C^ protein from 609 animal samples comprising 29 species from 22 genera of the Order Carnivora; amongst these samples were 15 FSE cases. Our analysis revealed that FSE cases did not encode an identifiable disease-associated PrP polymorphism. However, all canid PrPs contained aspartic acid or glutamic acid at codon 163 which we propose provides a genetic basis for observed susceptibility differences between canids and felids. Among other carnivores studied, wolverine (*Gulo gulo*) and pine marten (*Martes martes*) were the only non-canid species to also express PrP-Asp163, which may impact on their prion diseases susceptibility. Populations of black bear (*Ursus americanus*) and mountain lion (*Puma concolor*) from Colorado showed little genetic variation in the PrP protein and no variants likely to be highly resistant to prions in general, suggesting that strain differences between BSE and CWD prions also may contribute to the limited apparent host range of the latter.

## Introduction

The fatal, neurodegenerative prion diseases, also known as transmissible spongiform encephalopathies (TSEs) have been detected in a wide range of species. These diseases cannot develop, and the disease agent does not replicate, without the expression of the prion gene (*PRNP*) encoding the prion protein PrP [1], [2]. In a healthy animal, cellular PrP (PrP^C^) is highly expressed on the neuronal cell surface, but many other cell-types also produce PrP^C^. Following infection, normal PrP^C^ is converted into abnormal PrP^Sc^ which accumulates as a range of protease-sensitive and –resistant isoforms. This process involves a change in protein conformation but no modification of amino acid sequence. PrP^Sc^ then accumulates often as oligomers and amyloid fibrils in lymphoid and neuronal tissues [3], [4]. Mature PrP^C^ is a glycoprotein of approximately 210 amino acids which is well conserved in structure and sequence throughout the Mammalia. Within species, sequence variants of *PRNP* may give the prospective host a different risk of succumbing to prion diseases. For example it is very difficult to transmit the prion disease classical scrapie to sheep homozygous for a PrP^C^ variant with arginine at codon 171, whereas sheep homozygous for PrP^C^ with valine at codon 136 will almost always succumb to classical scrapie [5]. Similar associations exist in other species and are used to assign a prion disease risk to a specific *PRNP* genotype. In man, one particularly strong association with prion disease risk has been described for a codon 129 polymorphism and this polymorphism may be under balancing selection in the human population, with homozygotes for either allele more susceptible to TSEs than heterozygotes [6]. It has been suggested that similar selection may also act on *PRNP* in other species, such as sheep [7]. Sequence variation may also affect normal proteolytic cleavage of PrP^C^ which generates two fragments N1 and C1 [8]; importantly *in vivo* C1 appears not to be converted into PrP^Sc^ and may act as a dominant-negative modulator of infection [9]. Some prion diseases transmit between species but this process is relatively inefficient between more distantly related species and this so-called “species barrier” may be to a large extent a PrP^C^ sequence “compatibility” barrier, caused by species specific differences at key amino acids interfering with the PrP conversion and agent replication process [10], [11].

Prion diseases are typically observed in man and ruminants, but outbreaks of feline spongiform encephalopathy (FSE) in various feline species [12] and transmissible mink encephalopathy (TME) in farmed mink (*Neovision* (*mustela*) *vison*) [13] have shown that species of the Order Carnivora can be infected with prions under natural conditions, i.e. through contaminated feed. Scrapie in domestic sheep and goats, bovine spongiform encephalopathy (BSE) in cattle and chronic wasting disease (CWD) in deer species are sources of prion infectivity that may represent a risk to carnivores. In particular, predators and scavengers are exposed to CWD in the USA and Canada [14], [15]. In Europe, where scrapie has been endemic for well over two centuries, wild carnivores may feed occasionally on scrapie-infected livestock.

If spontaneous or transmitted prion diseases exist in prey in other regions of the world, genetically more resistant predators and scavengers may have been advantaged. If so, a signature of this process may be detectable in the PrP sequence of species such as large cats, wolf (*Canis lupus*) or wolverine (*Gulo gulo*). Experimentally, CWD has been transmitted to ferrets [16], [17] and domestic cats [18], but clinical disease was only achieved after intracerebral inoculation and not through ingestion. Importantly, repeated interspecies transmission did change the host range of the CWD isolate so that hamsters became susceptible after a number of passages of the infectious agent in ferrets [17]. This change of tropism by passage through new species has also recently been shown for BSE [19] and may represent an additional risk of prion infection in carnivores. Scavenger rodents are susceptible to challenge with CWD [20], and they in turn are prey for a number of small carnivore species including those not necessarily feeding on CWD-infected carrion. However, testing for PrP^Sc^ in mammalian carnivores from CWD-affected areas did not confirm any prion positive animals and suggested that cross-species transmission is not yet frequent [21].

BSE represented a true risk to carnivores in Europe. There were 89 confirmed cases of FSE in domestic cats in the UK (1990–2001) [22], [23] and four cases in Europe (1995–2003) [24], [25], [26]. Additionally, 23 captive feline predators originating from UK zoo collections had confirmed FSE [27]. Moreover, two cases not directly linked to UK FSE have been reported, one in an Asiatic golden cat (*Catopuma temmincki*) from Australia [28], the other in a cheetah (*Acinonyx jubatus*) born to a French FSE case but developing disease in a German zoo [29]. Quite the opposite is true for canine species, which have never shown prion disease. Surprisingly, *PRNP* genotypes have never been established for FSE cases, so that important questions remain regarding genetic susceptibility or disease transmission in carnivores.

The current availability of *PRNP* sequences shows a very uneven distribution amongst all mammals. Probably reflecting the occurrence of prion disease, approximately 50% are from the two Orders Artiodactyla and Primates, with only 2% from the Order Carnivora [30], [31]; the genetic variation of *PRNP* within this order is mostly unknown due to a lack of comprehensive analyses of wild populations or companion animals. Three species derived from farmed populations have been analysed in larger number: 135 Arctic foxes (*Vulpes lagopus*) revealed three PrP protein variants [32], whereas 56 mink and 20 raccoon (*Procyon lotor*) samples uncovered three and four variants, respectively [33], [34], [35].

Analysing the molecular and population genetics of *PRNP* should enable a genetically-based assessment of the risk for carnivores that may become or already are exposed to prion diseases. This study has shown PrP^C^ protein fragments in several carnivore species which are comparable to those found in other mammals. We have revealed a low degree of genetic variation within species but a number of remarkable interspecies differences for the over 600 *PRNP* sequences from a wide range of analysed carnivores. We conclude that the genetic uniformity of *PRNP* in American black bear (*Ursus americanus*) and mountain lion (*Puma concolor*) could result in homogeneous susceptibility to a compatible prion strain within these species - should such a strain emerge - unless non-*PRNP* genes associated with resistance are present.

## Results

### PrP^C^ Protein Processing

**Figure 1.Western:**
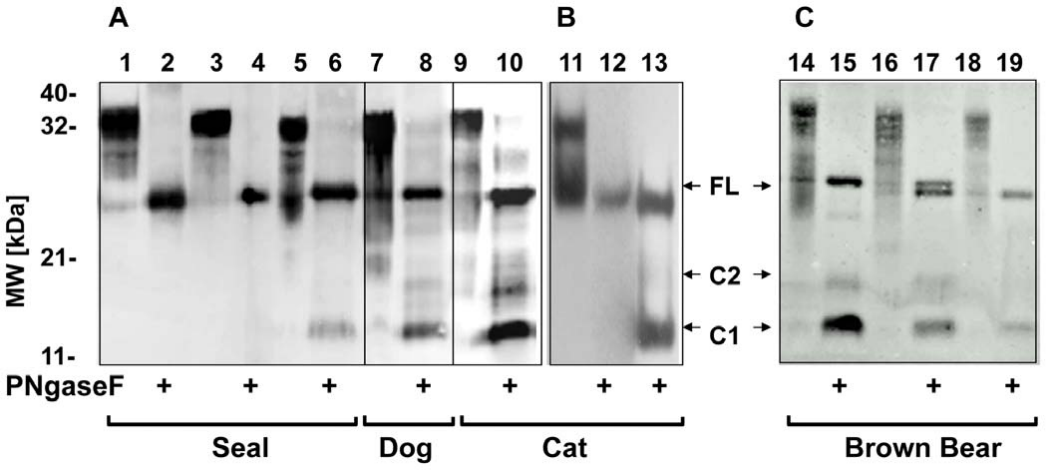
blot analysis of PrP^C^. Brain or spinal cord tissue was homogenized and analysed by Western blot as described in *Material & Methods* with various anti-PrP antibodies. Hooded Seal, one sample lanes 1–6; domestic dog, one sample lanes 7–8; domestic cat, two samples, 9–10 and 11–13; Brown bear, three samples 14–15, 16–17, 18–19. Untreated PrP^C^: lanes 1,3,5,7,9,11,14,16,18. Deglycosylated PrP^C^ (+): lanes 2,4,6,8,10,12,13,15,17,19. Detection using antibodies with epitopes N-terminal to α-cleavage site: SAF32 (lanes 1–2) or F11 (lane 12), with epitope in α-cleavage site: 3F4 (lanes 3–4), with epitopes C-terminal to α-cleavage site: Bar224 (lanes 5–10, 14–19), 6H4 (lane 11) or BC6 (lane 13).

We analysed neuronal tissue from representatives of four Families: Phocidae, Ursidae, Felidae and Canidae by Western blot to verify that similar PrP^C^ isoforms to those described for human and ruminant tissue are present in carnivores. All expressed glycosylated PrP^C^ of the expected molecular mass range, which after de-glycosylation shifted to an apparent molecular mass of 27 kDa (full-length PrP^C^) and 17 kDa (C1 fragment) (Fig. 1). All samples contained significant amounts of C1 fragment, representing 20–50% of total PrP^C^ expressed in these tissues. In the bear, cat and dog tissue a third band was visible of intermediate molecular mass. This corresponds most likely to the C2 fragment, which has previously been described in healthy tissue [8]. As expected, C1 and C2 were not detected by antibodies SAF32 and F11 against N-terminal PrP^C^ epitopes (Fig. 1, lanes 1, 2, 12). In brown bear (*Ursus arctos*) two PrP^C^ protein variants with different apparent molecular mass were expressed at similar level and both were proteolytically processed; the size variants reflected the two *PRNP* open reading frames of 249 and 257 amino acids (Table 1). We conclude that proteolytic processing of carnivore PrP^C^ is not fundamentally different to primates or artiodactyls.

**Table 1:** Frequencies of amino acid polymorphisms.

Species (Number of alleles tested)	Codon	Amino acid		Frequency	Amino acid	Frequency
Dog, *C. familiaris* (182)	**101**	**S**		0.66	**G**	0.34
	**163**	**D**		0.68	**E**	0.32
	**196**	**T**		0.96	**I**	0.04
Wolf, *C. lupus* (108)	**101**	**S**		0.45	**G**	0.55
Coyote, *C. latrans* (40)	**101**	**S**		0.05	**G**	0.95
Hooded seal, *C. cristata* (48)	**7**	**G**		0.96	**S**	0.04
	**101**	**S**		0.04	**G**	0.96
	**196**	**T**		0.98	**I**	0.02
Harp seal, *P. groenlandica* (2)	**17**	**T**		0.50	**A**	0.50
Wolverines, *G. gulo* (40)	**37**	**G**		0.60	**A**	0.40
European Polecat, *M. putorius* (6)	**223**	**R**		0.67	**Q**	0.33
Golden Cat, *C. temminckii* (2)	**181**	**N**		0.50	**S**	0.50
	**Repeat Number**	**Frequency**	**Repeat number**	**Frequency**	**Repeat number**	**Frequency**
Brown Bear, *U. arctos* (82)	**4**	0.24	**5**	0.76	**6**	0.00
Hooded Seal, *C. cristata* (48)	**4**	0.00	**5**	0.98	**6**	0.02
Cat, *F. catus* (256)	**4**	0.06	**5**	0.94	**6**	0.00

### *PRNP* Open Reading Frame Haplotypes in Felidae

**Figure 2:**
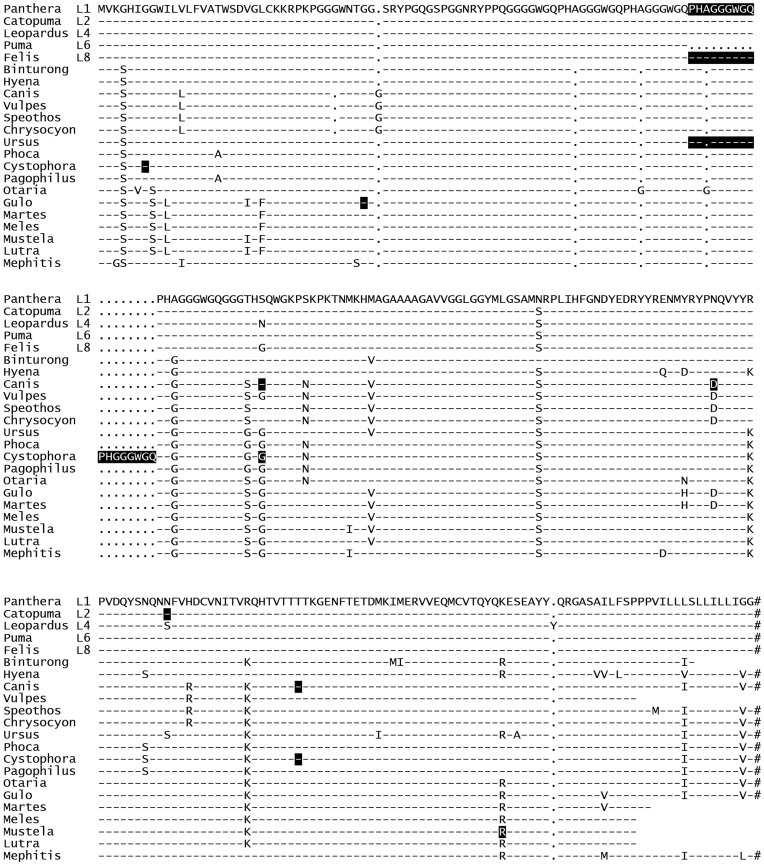
PrP protein sequence alignment. hyphen, identical amino acid to top sequence; dot, deletion; #, stop codon, end of coding region; L1–L8, cat lineages [34]. White letters on black background, these positions are polymorphic (see Table 1). Sequences that are not shown as full length were generated with DNA primers on the end of the open reading frame. The correct species sequence can therefore not be inferred for these amino acids. Panthera (L1): Asiatic lion (Panthera leo persica), jaguar (Panthera onca), tiger (Panthera tigris & Panthera tigris sumatrae), Persian leopard (Panthera pardus saxicolor) Catopuma (L2): Asiatic golden cat (*Catopuma temminckii*) Leopardus (L4): margay (*Leopardus wiedii*) Puma (L6): cheetah (*Acinonyx jubatus*), mountain lion (*Puma concolor*) Felis (L8): cat (Felis catus), Bengal cat (Felis catus x Prionailurus bengalensis (L7)) Binturong: binturong (*Arctictis binturong*) Hyena: spotted hyena (*Crocuta crocuta*) Canis: coyote (Canis latrans), gray wolf (Canis lupus), dog (Canis familiaris) Vulpes: red fox (*Vulpes vulpes*) Speothos: bush dog (*Speothos venaticus*) Chrysocyon: maned wolf (*Chrysocyon brachyurus*) Ursus: brown bear (*Ursus arctos*), Black bear (*Ursus americanus*) Phoca: common seal (*Phoca vitulina*) Cystophora: hooded seal (*Cystophora cristata*) Pagophilus: harp seal (*Pagophilus groenlandica*) Otario: Patagonian sea lion (*Otaria flavescens*) Gulo: wolverine (*Gulo gulo*) Martes: European pine marten (*Martes martes*) Meles: badger (*Meles meles*) Mustela: European polecat (*Mustela putorius*) Lutra: European otter (*Lutra lutra*) Mephitis: striped skunk (*Mephitis mephitis*).

The Family Felidae was represented by eight species with a total of 249 samples analysed. Felidae can be divided into eight lineages of cats [36], of which five were represented in our collection by at least one species. Eight different PrP variants could be defined based on amino acid differences and only one single amino acid polymorphism (Asn181Ser) was detected in one species, the Asiatic golden cat (Figure 2). We observed some segregation of haplotypes by lineage, for example all species of lineage 1 encoded asparagine at codon 142 whereas species of lineages 2, 4, 6 and 8 encoded serine at codon 142. The most divergent sequence of all Felidae came from the margay (*Leopardus wiedii*, lineage 4) which had a unique substitution with asparagine at position104 and a tyrosine insertion between codons 233 and 234.

**Figure 3:**
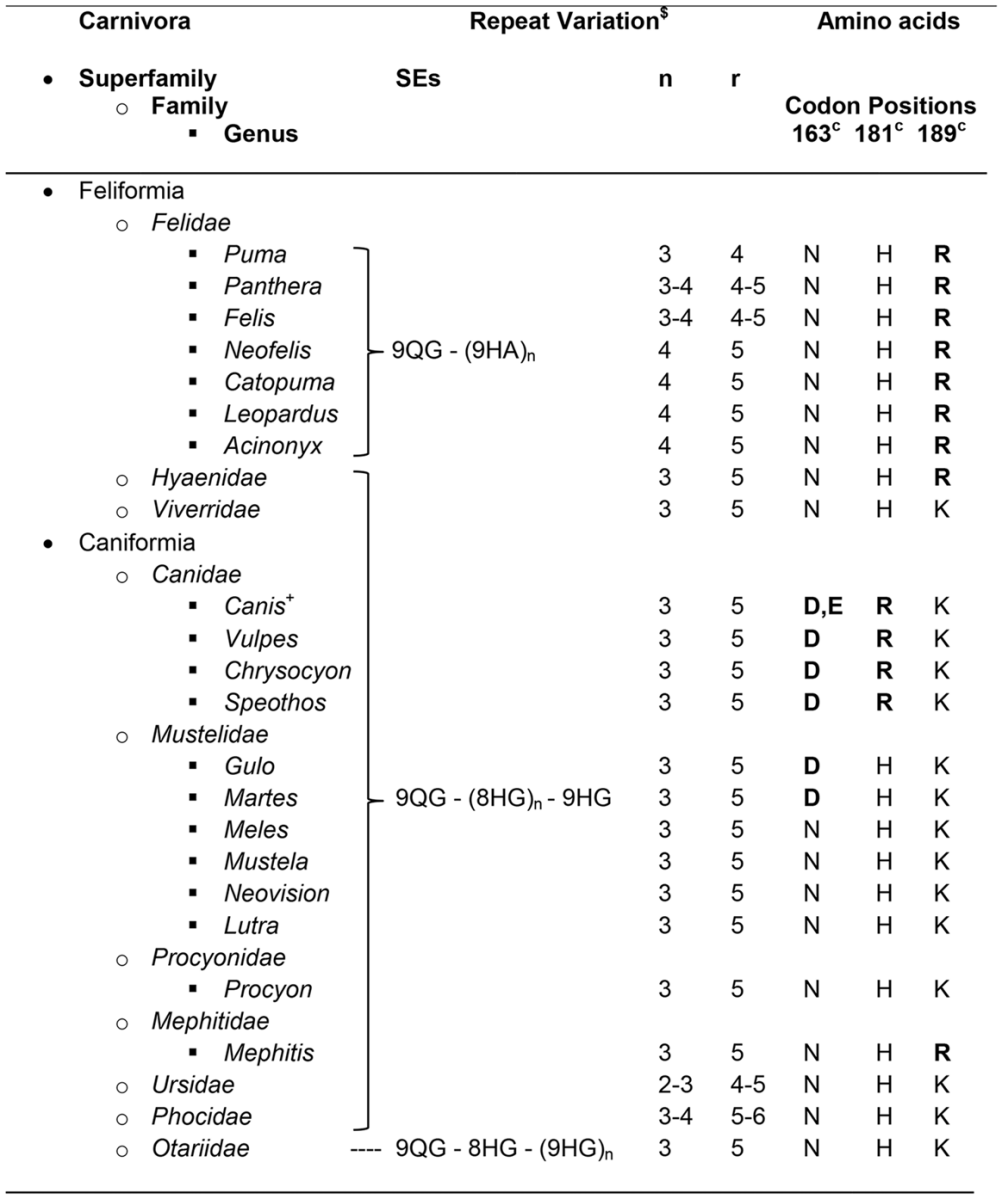
Particular sequence variations in PrP^C^ from species of the Order Carnivora. $ SEs: Sequence Elements, 9QG = nonapeptide PQGGGGWGQ, 9HA = nonapeptide PHAGGGWGQ, 8HG = octapeptide PHGGGWGQ, 9HG = nonapeptide PHGGGGWGQ; n, number of repeated SEs in brackets; r, number of total SEs (mode for all other species is five); Caniformia codon positions, equivalent codons for *Felidae* +3 when r = 5. (equivalent codons in *Primates* −4, in *Rodentia* −5). All other known *PRNP* sequences (>400): 98% N, 99% H, 99% K.

It had been shown previously that the characteristic glycine-rich octapeptide repeats in the N-terminal part of PrP have undergone mutational changes to alanine-containing nonapeptides in feline PrP [30], [37]. Our data confirms that this change is present in all species that were analysed from the Family Felidae. However, similar to many other mammals the repeat number varies between *PRNP* haplotypes. Whereas most had a total of five nonapeptide repeats, mountain lion PrP had only four. In domestic cats both variants appeared and in Old World lions it seems that repeat number segregates with subspecies: The Asiatic lion (*Panthera leo persicsa*) has five nonapeptide repeats and the African lion (*Panthera leo*) four [38]. It is however equally likely that these species are polymorphic in this protein domain. The alanine-containing nonapeptides appear to be unique to Felidae as they were not found in the closely related Families Viverridae (civet family) and Hyaenidae (hyena family) represented here by the binturong (*Arctictis binturong*) and the spotted hyena (*Crocuta crocuta*), respectively (Figures 2, 3).

### *PRNP* Allele Frequencies in Felidae

To explore the frequencies of the most common PrP variants, we analysed large sample groups of mountain lions (n = 101) from Colorado, USA and domestic cats (n = 118) from Europe and North America. Most surprising was the total lack of any sequence variation of *PRNP* in the 101 mountain lion samples. We analysed two microsatellite loci (FCA8 and FCA96, [39]) and could show that there was as expected a varied genotype distribution for these loci (data not shown). To guarantee a wide genetic background for the domestic cats we collected from various locations in Europe (United Kingdom, Switzerland) and America (USA). The 118 cat samples revealed no single amino acid polymorphisms but variation in the nonapeptide repeat number, either five or four. The 4-repeat haplotype frequency was 6% overall and it was present in four out of five sampled populations. There were seven synonymous polymorphisms in three high frequency haplotypes and several minor haplotypes (data not shown). The most common haplotype was the same for all four sample groups and only domestic Bengal cats showed different frequency patterns. All our sequences, including ten cats from Switzerland, were different in codon 246 (Gly) to the published sequence (Arg) of a Swiss domestic cat [37].

### *PRNP* Genetics in FSE Cases

Although all domestic cats that were analysed for *PRNP* showed the same amino acid sequence we reasoned that it was possible that the FSE cases encoded rare variations which may have increased their susceptibility. Of the 89 UK FSE cases in domestic cats only nine were available for DNA analysis. These nine UK cases came from the first two years (1990/1991) of the BSE epidemic which recorded 24 cases and they include the index case. Representing 38% of the early phase they are likely to reveal whether or not these animals were more susceptible than others by carrying a specific *PRNP* polymorphism. Our analysis shows that the deduced PrP protein sequences from the nine FSE cases were not different from control samples collected either at the same time or recently. They were all homozygous for the five nonapeptide repeat variant.

FSE cases from six cheetahs located in France (five) and Germany (one) [29], [40], [41], [42] were genotyped and no amino acid polymorphism was detected. We compared the deduced cheetah sequences to PrP from Asiatic lion, mountain lion, tiger (*Panthera tigris* and *Panthera tigris sumatrae*), golden cat, and domestic cat, all of which are susceptible to FSE (via the BSE infectious agent). There are only two codon differences between these six species, at codons 104 (Gly/Ser) and 142 (Asn/Ser). The cheetah PrP contains Ser104-Ser142, identical to puma and golden cat. Lion and tiger are Ser104-Asn142 and domestic cat Gly104-Ser142. No other tissues of FSE cases from zoo collections have been available for genetic analysis.

### *PRNP* Open Reading Frame Haplotypes in Canidae

Prion diseases have never been reported for canine species. Whether this was due to a lower exposure than for felids during the United Kingdom BSE epidemic [43] remains an open question, but it is likely that fundamental differences in genetic susceptibility exist between these Families [44], [45]. Genotyping 170 samples from five species of the Family Canidae revealed five PrP protein variants, of which three were the result of polymorphisms in dogs at codons 101 (Gly/Ser), 163 (Asp/Glu) and 196 (Thr/Ile). No variation in the octapeptide repeat number was seen in these PrP sequences. The substitution of Asn with Asp in codon 163 was highly characteristic for the canidae family and not observed anywhere else with the exception of wolverine and marten PrP (see below and Figure 3). The PrP variant with Glu163 had a high frequency in our dogs (32.4%, n = 91) but in gray wolves it was either absent or had a frequency of less than 4% (at 99% probability) [46]. In contrast, the Ser101 variant was found with moderate to high frequencies in coyotes (*Canis latrans*), gray wolves (*Canis lupus*) and domestic dogs (5%, 45% and 66% respectively) (Table 1).

### Bear and Seal *PRNP*

Most bears are opportunistic omnivores that will occasionally take prey such as deer or feed on carcasses. Their close taxonomical relatives are seals which as marine mammals are unlikely to be exposed to prion agent from their prey. We analysed *PRNP* sequences from 139 bear and 26 seal (Phocidae & Otariidae) samples. Their sequences fitted into the Suborder Caniformia by sharing amino acids with Canidae and Mustelidae, but there were no exclusive amino acid substitution for these two Families together or on their own (Figures 2, 3). This relationship is also apparent from the polymorphisms: the hooded seal (*Cystophora cristata*) shared polymorphisms at codons 101 (Ser/Gly) and 196 (Thr/Ile) with the canines. Additionally, a six octapeptide repeat haplotype was found in one hooded seal and a four octapeptide repeat haplotype in the brown bear.

### *PRNP* in the Superfamily Musteloidea

Although no case of prion disease has been reported in a free-ranging individual of any species within this superfamily, mink develop TME in captivity and must therefore be regarded as carrying a susceptible *PRNP* genotype. Ferrets and raccoons are also susceptible to experimental challenges with prion infectivity. We added *PRNP* sequences from European otter (*Lutra lutra*), European polecat (*Mustela putorius*), European badger (*Meles meles*), pine marten (*Martes martes*) and wolverine as well as striped skunk (*Mephitis mephitis*) to the already available species in this superfamily. The wolverine and pine marten PrP^C^ sequences were exceptional with amino acids His159 and Asp163, as all other members of this Superfamily that have been sequenced encode Tyr159 and Asn163. We only studies a small number of individuals per species and would therefore only expect to find high frequency polymoprhisms such as the Arg/Gln substitution at codon 223 of the European polecat.

## Discussion

Prion diseases have not been observed in wild populations of carnivores, but some species of this order can become infected with prions and will develop clinical disease, prominent examples are FSE in cats and TME in mink. There is also no doubt that some carnivores are naturally exposed through their prey or carrion, such as to CWD infected deer and elk in the USA and Canada. However, there has been little research on the genetic susceptibility of carnivores to prion diseases and whether natural genetic variation in the prion gene is likely to influence transmission and susceptibility. In this study we have analysed the *PRNP* open reading frame sequences from a large number of species including FSE cases and evaluated *PRNP* sequence variability in wild populations.

Presence of PrP^C^ is essential for the development of TSEs and dog, cat and mink do express detectable levels of PrP^C^ [47], [48]. Our data confirm this and reveal that PrP^C^ in these species, including in bear and seal, is processed normally by proteolytic cleavage into fragments, which are equivalent to those seen in prion disease susceptible species such as sheep and man. Not only are these fragments produced but they also appear at ratios similar to other mammals; it remains to be shown whether different PrP^C^ fragment ratios between canine and feline species underpin the different susceptibilities.

The lack of PrP variants associated with FSE-infected cats and cheetahs indicated that these animals were not at higher genetic risk than other individuals of their species. It can therefore be inferred –possibly with the inclusion of the lions- that FSE risk in the feline species was not controlled by highly susceptible PrP variants. The opposite, that most cats are genetically resistant through the presence of a common, “protective” PrP allele that is different to the susceptible “wildtype” allele can also be rejected, as our analysis of 68 UK cats and 118 cats in total should have revealed with 99.9% probability any alleles with frequencies equal to or higher than 5% and 3%, respectively [46]; the 4-repeat allele is not frequent enough to fulfil this role although we cannot exclude that it may have some protective effect.

The small number of FSE cases may be a consequence of low exposure, low natural transmission probability and possibly a large degree of underreporting of cases from a population in the millions, but it is also possible that non-*PRNP* genes with protective phenotype are particularly advantageous in felines or that fixed amino acid changes in the feline wildtype PrP sequence make cats partially resistant to prions. Although it could be argued that the considerable number of FSE cases in the relative small number of large cats in zoo collections does not support this hypothesis, they may have been exposed to a larger dose of prion infectivity than their domestic counterparts. The addition of alanine into the N-terminal repeats represents a feline PrP^C^ specific change. It is difficult to predict its contribution to susceptibility. Although the repeats are not essential for replication of the agent or the PrP^C^ to PrP^Sc^ conversion [49], they can modulate disease phenotypes such as PrP^Sc^ accumulation and incubation periods [49], [50].

Cats may show average susceptibility or even partial resistance, in which case one has to ask whether the failure to find canine BSE cases has been a reflection of exposure, surveillance, diagnostics or genetics. There appears to be a strong case for a genetic/biochemical explanation because *in vitro* amplification experiments with canid PrP showed reduced conversion ability [44], [45]. By expanding the *PRNP* sequence analysis to large number of felids and canids we have shown that differences are consistently present in two regions of PrP: in codons 102^f^/99^c^, 110^f^/107^c^, 119^f^/116^c^ and in codons 166^f^/163^c^, 184^f^/181^c^ and 192^f^/189^c^. The substituted amino acids (Ser99^c^, Asn107^c^ and Val116^c^) in canids have all been found in other species that are susceptible to prions and are unlikely to explain the different BSE susceptibility between felids and canids. The three C-terminal substitutions may be more illuminating as they show specificity for the respective Families. The variation between Arg192^f^ and Lys189^c^ (with Lys the most commonly found amino acid in the equivalent position in other species) can be regarded as conservative and may lead to minimal structural change in the protein, nevertheless it is worth exploring whether PrP with Arg192 changes susceptibility of Felidae to prion disease. Both Asp163^c^ and Arg181^c^ appear to be conserved “signature” amino acids for all Canidae as they were present in five genera. These major substitutions of Asn with Asp or Glu and His with Arg in highly conserved positions [30] are close to a β–strand (Asp163) and loop (Arg181) structures that appear to be critical in PrP^C^ to PrP^Sc^ conversion (Figure S1) [51]. All three substitutions (Asp, Glu and Arg) result in changes of the charge distribution of PrP^C^ at cellular pH and may lead to different inter- and intramolecular interactions [52]; when Asn was replaced with Asp in the equivalent codon of mouse PrP^C^ (codon 158) in transgenic mice they were unable to produce PrP^Sc^ after prion challenge [53].

A thorough analysis of these and similar transgenic models in combination with *in vitro* studies of “signature substitutions” such as canine codon 163 or Arg192^f^ will eventually reveal the underlying molecular mechanisms of prion replication.

In contrast to the taxonomical relationship between Canidae and Mustelidae, the European wolverine and its close relative the Pine marten share the 163Asp substitution with canine PrP, whereas the omnivorous European badger, also a close relative to the wolverine does not. Hence, wolverines as predators of smaller ruminants and consumers of carrion may have similar genetic susceptibility as canids. The fixation of this asparagine in the wolverine and pine marten sequence is puzzling. How likely is it that 163Asp became fixed due to selection for PrP^C^ rather than by random genetic drift? Studies in sheep investigating the influence of preclinical prion diseases on fitness and reproductive are not conclusive in this regard [54]. Genetic drift is however supported by the fact that 163Asp is seen in PrP^C^ from an unrelated third species, the insectivorous little brown bat (*Myotis lucifugus, Access. No. BN000992*).

All animals from wild populations for which we analysed a larger number of samples (bear, wolf, mountain lion, seal) showed a very limited variation of their PrP^C^ sequences. No amino acid polymorphisms were found in *PRNP* of mountain lion and black bear, only one dimorphism each was seen in brown bears, Gray wolves, coyotes, wolverines and three dimorphisms were found in Hooded Seals. This is similar to wild populations such as red deer (*Cervus elephus*) and roe deer (*Capreolus capreolus*) [55], wapiti (*Cervus elaphus nelsonii*) (Goldmann, unpublished observation) and fallow deer (*Dama dama*) [56]. The sample sizes of any of our investigated groups were small so that allele frequencies are only estimates, but they should reveal alleles with lowest frequencies of 3–10% with 99% probability. In contrast to the large number of PrP^C^ variants maintained in livestock species such as sheep, sampling of companion animals such as cats and dogs did not appear to indicate an enhanced number of PrP^C^ variants compared to the wild carnivores. Whereas *PRNP* in sheep may be under balancing selection [7] it appears more likely that it is under purifying selection in free ranging populations but this needs to be formally proven. We observed a slightly larger number of PrP variants in the family Canidae than in the Felidae and this appeared to hold true for wild and domestic animals. It has been shown for different prion diseases that *PRNP* gene heterozygosity can reduce susceptibility and lengthen the incubation period, consequently it appears that as individuals and at the population level canine species like wolf and coyote may have a double genetic advantage (number of PrP variants and the preservation of variants with low conversion efficiency) over bear and puma with regard to overall CWD susceptibility.

This study has shown that FSE cases were not associated with *PRNP* genetics and that wild populations of carnivores have very little genetic variation in the *PRNP* gene which could result in unrestricted susceptibility to TSEs. Genetic uniformity of *PRNP* in American black bear and mountain lion could result in homogeneous susceptibility to a compatible prion strain within these species - should such a strain emerge -.

## Materials and Methods

### Ethics Statement

The use of samples from research studies was ethically approved. DNA samples acquired post mortem from road kill or when collected by the Parks and Wildlife agency for population management purposes do not fall under the restrictions of animal welfare laws. No animal was killed for the purpose of this study. Specific documentation: All Scandinavian samples were taken following ethical approval by the Ethical Committee on Animal Experiments, Uppsala, Sweden (C47/9 and C7/12). Seal samples were taken under permits issued by The Royal Norwegian Ministry of Fisheries and The Royal Danish Ministry of Foreign Affairs). Dog and cat sample use was approved by the Veterinary Ethical Review Committee of the University of Edinburgh, the Norwegian School of Vet. Sciences and Colorado State University Animal and Care Use Committee. The use of zoo samples was approved by the RZSS Research Committee, Edinburgh.

#### Samples

Presented as common name (species name, number of samples analysed, origin).

The samples used in this project were provided either from approved research projects, from zoo and museums archives or taken from road-kill cadavers. Wild population sampling:

Brown bear (*Ursus arctos*, 41, South-Central Scandinavia, collected from 2001–2007. http://skandulv.nina.no http://www.bearproject.info/sv/content); wolverine (*Gulo gulo*, 20, Sweden, collected during 2007 in Sarek National Park.

http://www.wolverineproject.se/The_swedish_wolverine_project/Welcome.html); grey wolf (*Canis lupus*, 54, South-Central Scandinavia); hooded seal (*Cystophora cristata*, 23, Greenland Sea; Mountain lion (*Puma concolor*, 101, Colorado, USA); black bear (*Ursus americanus*, 98, Colorado, USA), coyote (*Canis latrans*, 20, Colorado, USA);

Domestic, mostly mixed breed animals: Dog (*Canis familiaris*, 66, UK & 25, Norway), cat (*Felis catus* & *Felis catus* × *Prionailurus bengalensis*, 79, UK & Europe + 40, USA). Zoo archive: Sumatran tiger (*Panthera tigris sumatrae*,1, UK); tiger (*Panthera tigris*, 3, UK); jaguar (*Panthera onca*, 2, UK); Asiatic golden cat (*Catopuma temminckii*, 1, UK); margay (*Leopardus wiedii*, 1, UK); binturong (*Arctictis binturong*, 1, UK); Asiatic lion (*Panthera leo persica*, 5, UK); Patagonian sea lion (*Otaria flavescens*, 1, UK); bush dog (*Speothos venaticus*, 1, UK); striped skunk (*Mephitis mephitis*, 2, UK); Persian leopard (*Panthera pardus saxicolor*, 1, UK); harp seal (*Pagophilus groenlandica*, 1, Greenland Sea); common seal (*Phoca vitulina*, 1, UK); maned wolf (*Chrysocyon brachyurus*, 3, UK). Museums archives. European pine marten (*Martes martes,* 3, UK); European otter (*Lutra lutra*, 2, UK); red fox (*Vulpes vulpes*, 1, UK); badger (*Meles meles*, 3, UK); European polecat (*Mustela putorius*, 3, UK). FSE cases: domestic cat (*Felis catus*, 9, UK, Pearson et al, ref 10); cheetah (*Acinonyx jubatus*, 6, Germany, Eiden et al ref 27, France, Baron et al, ref 38); spotted hyena, (*Crocuta crocuta*, 1, USA, Hammond et al,(2012) Endocrinology 153∶1435).

GenBank sequence accession numbers: JX218943–JX218989.

#### Western blot

All tissue that was used for this analysis was frozen at −20°C or chilled instantly after removal from the carcass very shortly after death to minimize degradation [57] Briefly, brain samples were thawed and homogenized at 4°C in a Dounce all-glass homogenizer in lysis buffer (see Table S1). Samples were centrifuged to remove debris. Prior to SDS-electrophoresis samples were boiled in SDS-sample buffer (Invitrogen) in the presence of reducing agent (Invitrogen). Proteins were separated on 12% pre-cast gels. Samples were transferred onto poly(vinylidene difluoride) membrane and unspecific binding blocked by either 5% fat-free dried milk (seal samples) or western blocking reagent (Roche – cat samples). Membranes were washed with Tris buffered saline and incubated in primary antibody overnight. Seal samples were incubated in alkaline phosphatase conjugated secondary antibodies (GE Healthcare, UK) and proteins were visualized with a variable mode fluorescence imager (Typhoon 9200, GE Healthcare, UK) after incubation with the ALP substrate (ECF™, Western blotting reagent pack, GE Healthcare). Cat sample 2 was incubated in peroxidase conjugated secondary antibody (Stratech, UK) and manually developed after incubation with SuperSignal West Dura Extended Duration Substrate (Thermo Scientific).

#### Sequencing

PCR amplification of PrP from isolated genomic DNA (50–100 ng) was performed with Sigma JumpStart™ REDTaq® DNA polymerase, 200 µM (each) dNTPs (Roche, Switzerland) and 0.4 µM of each oligonucleotide primer. Various primers were located near or on the start and stop codon of the open reading frame (primer sequences available on request). PCR conditions were as follows: 3 min at 95°C followed by 40 cycles of 30 sec at 95°C, 30 sec at N°C and 1 min at 72°C and finally an elongation step of 10 min at 72°C, where N was selected by the choice of the primer pair, usually between 55–64°C. Purified PCR fragments were sequenced directly in both directions with BigDye® terminator v3.1 cycle sequencing kit using the PCR or additional internal primers. Purified reactions were run on an Applied Biosystems 3130 Genetic Analyzer (Applied Biosystems, USA). Electropherograms were analysed by eye and DNASTAR Lasergene Core Suite version 9. All polymorphic positions or sequences based on single samples were confirmed by repetition, in some cases with primers of increased species specificity.

## Supporting Information

Figure S1 **PrP protein sequence alignment (extended version of** **Figure 2** **).** Sequence alignment of new PrP protein sequences from this study compared to a selection of previously published species PrP (man, cattle, wapiti, mouse, rabbit, horse, pig).
(PDF)

Table S1 **Tissue preparation and Immunoblotting.** Details of method used in this study to prepare material for Western blot shown in Figure 1.
(PDF)
