# Supplementary material for: Genetic Predictions of Prion Disease Susceptibility in Carnivore Species Based on Variability of the Prion Gene Coding Region
Source: PLoS One. 2012 Dec 7;7(12):e50623. doi: 10.1371/journal.pone.0050623 (PMC3517517; doi:10.1371/journal.pone.0050623)
Supplement: Figure S1 — PrP protein sequence alignment (extended version of Figure 2 ). Sequence alignment of new PrP protein sequences from this study compared to a selection of previously published species PrP (man, cattle, wapiti, mouse, rabbit, horse, pig). (PDF) [file pone.0050623.s001.pdf]

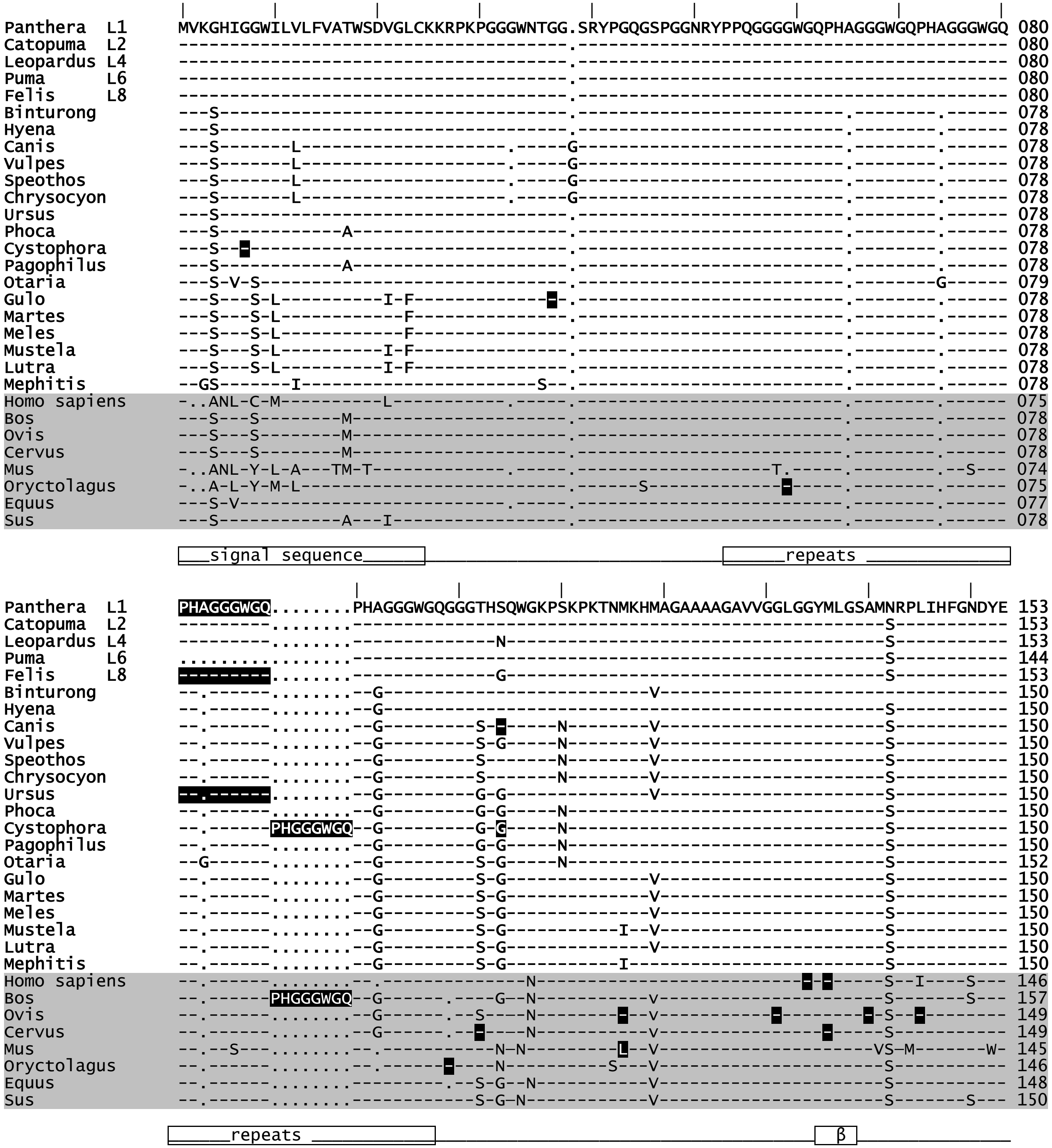

Panthera L1
Catopuma L2
Leopardus L4
Puma L6
Felis L8
Binturong
Hyena
Canis
Vulpes
Speothos
Chrysocyon
Ursus
Phoca
Cystophora
Pagophilus
Otaria
Gulo
Martes
Meles
Mustela
Lutra
Mephitis
Homo sapiens
Bos
Ovis
Cervus
Mus
Oryctolagus
Equus
Sus
MVKGHIGGWILVLFVATWSDVGLCKKRPKPGGGWNTGG.SRYPGQGSPGGNRYPPQGGGGWGQPHAGGGWGQPHAGGGWGQ 080
signal sequence
repeats
PHAGGGWGQ........PHAGGGWGQGGGTHSQWGKPSKPKTNMKHMAGAAAAGAVVGGLGGYMLGSAMNRPLIHFGNDYE 153
PHGGGWGQ
repeats
β

```
Panthera   L1   DRYYRENMYRYPNQVYYRPVDQYSNQNNFVHDCVNITVRQHTVTTTTKGENFTETDMKIMERVVEQMCVTQYQKESEAYY. 233
Catopuma   L2   --------------------------------------------------------------------------------. 233
Leopardus  L4   ---------------------------S----------------------------------------------------Y 234
Puma       L6   --------------------------------------------------------------------------------. 224
Felis      L8   --------------------------------------------------------------------------------. 233
Binturong       --------------------------------------K-------------------MI-------------R------. 230
Hyena           -----Q--D--------K------S------------------------------------------------R------. 230
Canis           ------------D-----------------R-------K-----------------------------------------. 230
Vulpes          ------------D-----------------R-------K-----------------------------------------. 230
Speothos        ------------D-----------------R-------K-----------------------------------------. 230
Chrysocyon      ------------D-----------------R-------K-----------------------------------------. 230
Ursus           -----------------K---------S----------K-----------------I----------------R-A----. 230
Phoca           -----------------K------S-------------K-----------------------------------------. 230
Cystophora      -----------------K------S-------------K-----------------------------------------. 230
Pagophilus      -----------------K------S-------------K-----------------------------------------. 230
Otaria          --------N--------K--------------------K----------------------------------R------. 232
Gulo            --------H---D----K--------------------K----------------------------------R------. 230
Martes          --------H---D----K--------------------K----------------------------------R------. 230
Meles           -----------------K--------------------K----------------------------------R------. 230
Mustela         -----------------K--------------------K----------------------------------R------. 230
Lutra           -----------------K--------------------K----------------------------------R------. 230
Mephitis        -----D-----------K-------------------------------------------------------R------. 230
Homo sapiens    --------H----------M-E---------------IK-----------------V-M---------I---ER--Q---. 226
Bos             --------H-----------------------------KE----------------I-M---------I----R--Q---. 237
Ovis            --------------------------------------K-----------------I-----------I----R--Q---. 229
Cervus          -----------------------N---T----------K-----------------I-M---------I----R------. 229
Mus             -------------------------------------IK-----------------V-M-----------------Q---. 225
Oryctolagus     ---------------------------S----------------------------I-----------I----Q--Q-A-. 226
Equus           --------------------SE----------------K-----------------V-----------I------Y--FQ. 228
Sus             ---------------------------S----------K-----------------V-MI--------I------Y---A. 230

                helix 1      β        helix 2                 helix 3

Panthera   L1   Q.RGAS.AILFSPPPVILLLSLLILLIGG# 260
Catopuma   L2   -.----.----------------------# 260
Leopardus  L4   -.----.----------------------# 261
Puma       L6   -.----.----------------------# 251
Felis      L8   -.----.----------------------# 260
Binturong       -.----.------------I-
Hyena           -.----.VV-L--------V-------V-#
Canis           -.----.------------I-------V-# 257
Vulpes          -.----.-------
Speothos        -.----.--------M---I-------V-# 257
Chrysocyon      -.----.------------I-------V-# 257
Ursus           -.----.------------I-------V-# 257
Phoca           -.----.------------I-------V-# 257
Cystophora      -.----.------------I-------V-# 257
Pagophilus      -.----.------------I-------V-# 257
Otaria          -.----.------------I-------V-# 259
Gulo            -.----.-V----------I-------V-# 257
Martes          -.----.-V------
Meles           -.----.-------
Mustela         -.----.-------
Lutra           -.----.-------
Mephitis        -.----.-M----------I-------L-# 257
Homo sapiens    -.--S-.MV---S------I-F--F--V-# 253
Bos             -.----.V----S------I-F--F--V-# 264
Ovis            -.----.V----S------I-F--F--V-# 256
Cervus          -.----.V----S------I-F--F--V-# 256
Mus             DG-RS-STV---S------I-F--F--V-# 254
Oryctolagus     -.-A-G.VL---S------I-F--F--V-# 253
Equus           -.----.VV---S---V--I-F--F--V-# 255
Sus             -.----.V----S------I-F-LF--V-# 257

                        GPI signal
```
