# Supplementary material for: Genetic Predictions of Prion Disease Susceptibility in Carnivore Species Based on Variability of the Prion Gene Coding Region
Source: PLoS One. 2012 Dec 7;7(12):e50623. doi: 10.1371/journal.pone.0050623 (PMC3517517; doi:10.1371/journal.pone.0050623)
Supplement: Table S1 — Tissue preparation and Immunoblotting. Details of method used in this study to prepare material for Western blot shown in Figure 1. (PDF) [file pone.0050623.s002.pdf]

Table S2 Tissue preparation and Immunoblotting

Tissue homogenising	Tissues were manually homogenised in lysis buffer (5 % NP-40 (v/v), 12.1 mM Sodium deoxycholate in PBS) with protease inhibitors (10 µM PMSF, 10 µM NEM or Complete Mini Tablets, Roche) to make a 10 % (w/v) homogenate. The homogenate was clarified by centrifugation at 2000 rpm at 4 ºC for 10 minutes, the supernatant was collected, flash frozen and stored at -20 °C until further analysis.
Deglycosylation	Brain homogenate (10 % w/v) was denatured at 100 ºC for 10 minutes and incubated with 0.125 U of peptide N-glycoside F (PNGase F kit, New England Biolabs) for 2 hours at 37 ºC according to manufacturer's instructions. Deglycosylated protein was isolated using methanol precipitation and stored at -20 ºC. Before immunoblotting, the protein was pelleted by centrifugation at 10,400 g for 10 minutes. Prior to electrophoresis, samples were boiled directly in NuPAGE (Invitrogen) sample buffer supplemented with a reducing agent (Invitrogen).
SDS PAGE	Deglycosylated protein was denatured at 70 ºC for 10 minutes and separated by 12% NuPAGE Bis-Tris gels (Invitrogen) or 12% Criterion gels (BioRad). Molecular markers spanning 20-220 kDa were used for size reference (MagicMarker XP Western protein Standard, Invitrogen) and electrophoresis was performed in an Xcell SureLock tank at 150 V for 1 hour using a NuPAGE kit (Invitrogen) or with the BioRad Criterion system (BioRad).
Immunoblotting	Proteins were transferred onto poly(vinylidenedifluoride) membranes (Millipore or GE Healthcare) at 25 V for 1 hour, after which the membranes were washed with TBS (50 mM Tris, 150 mM NaCl, pH 7.5). The membranes were blocked using 1 % (v/v) blocking solution for 1 hour at room temperature with agitation followed by incubation with anti-PrP antibodies diluted in 0.5 % (v/v) blocking solution under the same conditions. Membranes were washed with TBST (0.1 % Tween 20 in TBS) followed by 0.5 % (v/v) blocking solution. The membranes were incubated in horseradish-peroxidase-conjugated rabbit anti-mouse (Stratech, UK) or ALP conjugated goat-anti-mouse IgG (BioRad) diluted at 1:10000 in 0.5 % (v/v) block for 75 minutes. The membranes were washed in TBST and proteins were visualized using activated chemiluminescence (SuperSignal West Dura Extended Duration Substrate, Thermo Scientific) Lumi-Film Chemiluminescent Detection Film (Roche) or fluorescence (ALP substrate, ECF, GE Healthcare), recorded with a variable mode imager (Typhoon, GE Healthcare), when secondary antibodies labeled with ALP were used.
